# Delayed Recompression for Decompression Sickness: Retrospective Analysis

**DOI:** 10.1371/journal.pone.0124919

**Published:** 2015-04-23

**Authors:** Amir Hadanny, Gregori Fishlev, Yair Bechor, Jacob Bergan, Mony Friedman, Amit Maliar, Shai Efrati

**Affiliations:** 1 The Sagol Center for Hyperbaric Medicine and Research, Assaf Harofeh Medical Center, Zerifin, Israel; 2 Sackler School of Medicine, Tel-Aviv University, Tel-Aviv, Israel; 3 Research and Development Unit, Assaf Harofeh Medical Center, Zerifin, Israel; 4 Sagol School of Neuroscience, Tel-Aviv University, Tel-Aviv, Israel; University of Washington, UNITED STATES

## Abstract

**Introduction:**

Most cases of decompression sickness (DCS) occur soon after surfacing, with 98% within 24 hours. Recompression using hyperbaric chamber should be administrated as soon as feasible in order to decrease bubble size and avoid further tissue injury. Unfortunately, there may be a significant time delay from surfacing to recompression. The time beyond which hyperbaric treatment is non effective is unclear. The aims of the study were first to evaluate the effect of delayed hyperbaric treatment, initiated more than 48h after surfacing for DCS and second, to evaluate the different treatment protocols.

**Methods:**

From January 2000 to February 2014, 76 divers had delayed hyperbaric treatment (≥48h) for DCS in the Sagol center for Hyperbaric medicine and Research, Assaf-Harofeh Medical Center, Israel. Data were collected from their medical records and compared to data of 128 patients treated earlier than 48h after surfacing at the same hyperbaric institute.

**Results:**

There was no significant difference, as to any of the baseline characteristics, between the delayed and early treatment groups. With respect to treatment results, at the delayed treatment divers, complete recovery was achieved in 76% of the divers, partial recovery in 17.1% and no improvement in 6.6%. Similar results were achieved when treatment started early, where 78% of the divers had complete recovery, 15.6% partial recovery and 6.2% no recovery. Delayed hyperbaric treatment using US Navy Table 6 protocol trended toward a better clinical outcome yet not statistically significant (OR=2.786, CI95%[0.896-8.66], p=0.07) compared to standard hyperbaric oxygen therapy of 90 minutes at 2 ATA, irrespective of the symptoms severity at presentation.

**Conclusions:**

Late recompression for DCS, 48 hours or more after surfacing, has clinical value and when applied can achieve complete recovery in 76% of the divers. It seems that the preferred hyperbaric treatment protocol should be based on US Navy Table 6.

## Introduction

Decompression sickness syndrome (DCS) is caused by microbubbles forming in blood vessels or tissues during a reduction in environmental pressure (decompression). The bubbles are formed due to supersaturation when the rate of pressure reduction exceeds the rate of inert gas (mostly nitrogen, but occasionally helium) washout from tissues[1]. Bubbles have mechanical, embolic and biochemical effects with manifestations ranging from none to fatal [2]. Large bubbles, when formed, cause mechanical distortion of tissue and/or tissue hypoxia due to vascular obstruction. However, intravascular microbubbles may culminate in secondary tissue damage cascade including impaired endothelial function and an inflammatory reaction mediated by activated leukocytes, platelets and vasoactive compounds [3, 4].

By reducing bubble volume and hastening inert gas elimination, recompression therapy with hyperbaric treatment remains the main therapy for DCS. The most common hyperbaric protocol used is based on US Navy Treatment Table 6, started as early as possible after surfacing. In case of residual manifestations after the first hyperbaric treatment, additional sessions are recommended with shorter treatment duration (60–90 minutes each) and lower pressure (2–2.8 ATA). The outcome of hyperbaric therapy varies with complete resolution reported in 13–63% of patients suffering from severe DCS, and in 73–100% of patients with mild-moderate DCS [5–8].

In most cases of DCS, the symptoms appear immediately upon or soon after surfacing, where in 98% of the cases the clinical presentation is within the first 24 hours. However, in some cases there is delay and the symptoms are first presented more than 24 hours after surfacing. The late presentation of DCS is more common in divers having airplane flight or ascending to high altitude a day or two after the dive [9]. The exact incidence of late DCS (>48 hours) is unknown. In most published DCS case series, very few patients were treated later than 24 hours after surfacing [2, 4, 5]. Moreover, there may be a significant time lag between symptoms presentation and recompression in a hyperbaric chamber.

The significance of time to recompression (TTR) is controversial. Early studies suggested early recompression improved the clinical outcome, however recent studies showed TTR had very little effect on clinical recovery [5, 6, 8]. Moreover, the time beyond which hyperbaric treatment isn’t effective has not yet been determined.

Early hyperbaric treatment improves the outcome by decreasing bubble size and avoiding further tissue injury[10]. Hyperbaric treatment at the late DCS phase may still reduce the bubbles size, especially the micro-bubbles that can remain in tissues, blood and lymphatic vessels for prolonged durations of time. The expected clinical improvement by hyperbaric treatment can also be related to its capacity to deliver more oxygen to ischemic tissues and to its anti-inflammatory properties [11–16].

The primary objective of this study was to evaluate the effect of delayed hyperbaric treatment initiated more than 48 hours after surfacing on short term clinical outcome of DCS. The clinical outcomes of the delayed hyperbaric treatments were compared to early treatments given at the same hyperbaric unit. The secondary objective was to evaluate the different hyperbaric protocols used for delayed decompression.

## Methods

From January 2000 to February 2014, 204 divers suffering from DCS were treated in the institute of Hyperbaric Medicine, Assaf Harofeh Medical Center, Israel.

Recompression tables were decided by the physician on-site. In case of partial recovery, additional hyperbaric oxygen treatment (HBOT) sessions were given until the patient fully recovered or until no further improvement observed.

Diagnosis was based on clinical symptoms, signs and diving history. DCS was classified as type 1 or 2 accordingly. Arterial gas embolism cases were excluded from the study.

In addition, the cases were classified into mild, moderate and severe categories according to the symptoms and physical examination. Mild cases included skin, musculoskeletal or constitutional symptoms (malaise, fatigue, headaches and inattention). Divers with subjective limb numbness, severe musculoskeletal symptoms, pulmonary symptoms or mild chest pain were categorized as moderate. Cases were categorized as severe if one or more of the following was present: focal objective hypoesthesia, focal weakness, ear and balance symptoms, visual symptoms, urinary incontinence and pulmonary chokes. Clinical outcome was examined on follow up evaluation 10–14 days post treatment.

The data were collected retrospectively from medical records and included age, sex, diving experience, maximal depth, DCS possible cause, DCS type, symptoms, time from surfacing to symptoms onset, time from surfacing to recompression, recompression table used, additional treatment after the first recompression and treatment outcome. Total dive time was not included in the analysis due to lack of objective data logs in many of the cases.

The divers were divided into two groups: early recompression group (<48 hours) and delayed recompression group (≥48 hours from surfacing). The delayed group was further divided based on the time lag from surfacing to symptoms onset (12 hours was set as threshold).

Approval from the Helsinki Ethics Committee of Assaf Harofeh Medical Center was obtained for retrospective analysis of all cases used in this study. No written consent was needed according to the local ethics regulations. The study was registered in the US National Institute of Health Clinical Trails registry. (https://www.clinicaltrials.gov/ct2/show/NCT02243345).

### Statistical analysis

Data are expressed as mean ± SD for parametric variables and frequencies and percentages for nonparametric variables. Clinical outcome was used as an ordinal dependent variable (no recovery, partial recovery or full recovery) as well as binary nominal variable (full recovery or sequale). Univariate analysis was performed using Chi-Square/Fisher’s exact test to identify significant variables (P<0.05). Numeric variables analysis was performed using independent student t-test. Forward stepwise logistic regression was performed to control for potential confounders and to determine independent predictors for clinical outcome. In this model, highly intercorrelated independent variables (r>0.7) were avoided. Odds ratios (OR) and 95% confidence intervals (95% CI) were calculated. Methods were performed using the SPSS v.21 software.

## Results

**Fig 1:**
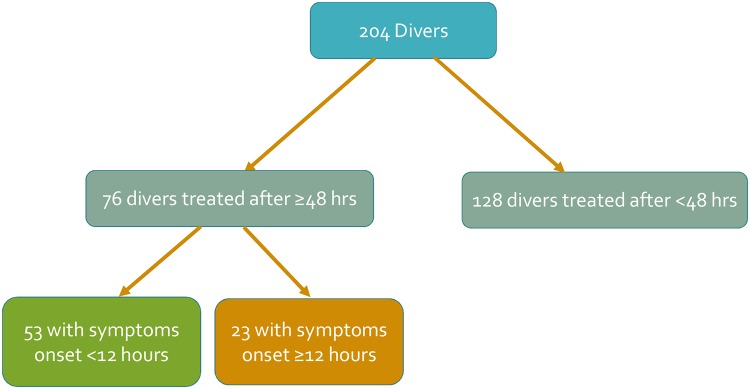
Flow chart describing study groups. * The divers were divided into early recompression group (<48 hours) and delayed recompression group (≥48 hours from surfacing). The delayed group was further divided by the time to symptoms onset.

A total of 204 divers treated for DCS at the hyperbaric institute of Assaf Harofeh medical center were included. In 76 divers the hyperbaric treatment was delayed for more than 48 hours after surfacing (the delayed group) while in 128 divers treatment started within 48 hours after surfacing (the early group). Fifty three (69.7%) divers in the delayed group had early symptoms onset (<12 hours) and twenty three (30.3%) had late symptoms onset (>12 hours), (Fig 1).

### Baseline characteristics

Baseline patients’ characteristics are summarized in Tables 1 and 2. There was no significant difference between the delayed and early treatment groups with respect to age, sex, diving experience, maximal diving depth, gas mixture.

**Table 1:** Patients' Baseline characteristics: Early (<48 hours) and Delayed (≥48 hours) recompression groups.

	0–48 Hours	≥48 hours			Early/Delayed Significance	Symptoms groups significance
	Total (n = 128)	Total (N = 76)	Symptoms onset ≤12 hrs	Symptoms onset >12hrs		
***Age***	32.9±11	32.7±9	33.5±8	30.9±11	0.894	0.281
***Sex***					0.908	1
Males	115 (83%)	56 (86%)	45 (85%)	19 (82%)		
Females	24 (17%)	9(14%)	8(15%)	4 (17%)		
***Diving Experience***					0.547	0.441
Open Water or less	19 (15%)	9 (12%)	5 (10%)	4 (17%)		
Advanced Open Water or higher	109 (85%)	67 (88%)	48 (90%)	19 (83%)		
***Maximal Diving Depth***	28±7	28±8	28±6	26±8	0.864	0.148
***Gas Mixture***						
Air	106 (83%)	66 (86%)	46 (87%)	20 (87%)	0.444	1
Nitrox/Trimix	22 (17%)	10 (14%)	7 (13%)	3 (13%)		

Delayed group was further divided by symptoms onset (≤12 and >12 hours from surfacing)

**Table 2:** DCS and recompression characteristics: Early (<48 hours) and Delayed (≥48 hours) recompression groups’ recompression characteristics.

	<48 hours	≥48 hours			Early/Delayed Significance.	Symptoms groups Significance
	(n = 128)	Total (N = 76)	Symptoms onset ≤12 hrs	Symptoms onset >12hrs		
***DCS***					0.502	0.749
Type I	19 (15%)	14 (18%)	9 (17%)	5 (22%)		
Type II	109 (85%)	62 (82%)	44 (83%)	18 (78%)		
**Severity**					0.939	0.438
Mild	28 (22%)	18 (24%)	11 (21%)	7 (30%)		
Moderate	71 (55%)	42 (55%)	29 (55%)	13 (56%)		
Severe	29 (23%)	16 (21%)	13 (24%)	3 (13%)		
***Probable Cause of DCS***					0.05*	0.156
Rapid ascent	34 (27%)	18 (24%)	16 (33%)	2 (10%)		
Invalid residual nitrogen time	57 (44%)	28 (36%)	19 (40%)	9 (43%)		
Repeated descents	5 (4%)	1 (1%)	0	1 (5%)		
Flight	0 (0%)	5 (6%)	3 (6%)	2 (9%)		
Unknown	24 (19%)	17 (22%)	10 (21%	7 (33%)		
***Time to symptoms onset (hours)***	4±6	8.7±11	2.3±3	23±6	0.001*	**<0.0001**
***Time to recompression (hours)***	19±11	93±90	93±99	93±67	<0.0001*	0.984
***Recompression table***					<0.0001*	0.762
US Navy Table 5	4 (3%)	2 (3%)	1 (2%)	1(4%)		
US Navy Table 6	108 (84%)	46 (60%)	31 (58%)	15 (65%)		
2 ATA	2 (2%)	27 (35%)	20 (38%)	7 (31%)		
CX30	14 (11%)	1 (1%)	1 (2%)	0		
**Adjunctive sessions**						
Patients treated	58 (45%)	41 (54%)	32 (60%)	9 (39%)	0.249	0.132
Number of sessions	1±3	1±1	1±1	1±1	0.251	0.07

Delayed group was further divided by symptoms onset (≤12 and >12 hours from surfacing).

The time lag from surfacing to onset of symptoms was longer in the delayed group than in the early group (8.7±11 vs. 4±6 hours, T = -3.936, df = 67, p<0.0001) (Table 2). Divers in the delayed group who experienced early symptoms onset had no significant differences in any of their baseline characteristics nor in their DCS severity compared to those who had late symptoms onset (Table 2).

### DCS Symptoms

The delayed group had more constitutional symptoms (93.5% vs. 83%, p = 0.033), chest pain (14.5% vs. 5.5%, p = 0.04) and no bladder dysfunction (0% vs. 6%, p = 0.027) compared to the early group. The total subjective and objective neurological symptoms were similar in both groups (p = 0.247, p = 0.405) (Table 3). There were no significant differences between groups with respect to DCS type and severity (Table 2).

**Table 3:** Symptoms distribution: Early (<48 hours) and Delayed (≥48 hours) recompression groups’ symptoms distribution.

		Early <48 hrs	Late >48 hrs	Significance
Subjective neurological symptoms		75 (59%)	38 (50%)	0.247
Neurological signs		34 (26%)	16 (21%)	0.405
	Focal Hypoesthesia	12 (9%)	7 (9%)	0.969
	Ataxia	2 (1.5%)	3(4%)	0.364
	Focal Weakness	4 (3%)	4 (5%)	0.474
	Vision loss	6 (5%)	0	0.086
	Hearing loss	2 (1.5%)	2 (2.5%)	0.63
	Incontinence	8 (6%)	0	*0.027
Chest pain		7 (5.5%)	11 (14.5%)	*0.04
Arthralgia		52 (40%)	30 (39%)	0.884
Constitutional symptoms		106 (83%)	71 (93.5%)	*0.033

The severity of the presenting symptoms could be predicted by the time to symptoms onset (B = 0.068, p = 0.021, OR = 1.071 CI 95% [1.010, 1.135]) but not by age, sex, maximal depth, gas type, diving experience, probable cause of DCS. However, the time from surfacing to symptoms presentation was negatively correlated with age (i.e. the older the patient, the faster onset of symptoms), yet it was not fully significant (coefficient = -0.2, p = 0.06).

### DCS probable cause

Based on the diving profile analysis, the probable causes for DCS were invalid residual nitrogen time (36.8% in the delayed group vs. 44.5% in the early group), rapid ascent (23.7% in the delayed group, vs. 26.6% in the early group) and unclear in 22.4% and 18.8% (delayed and early groups respectively), (*χ* ^2^ = 14.02, p = 0.05) (Table 2). Multiple comparisons Chi-square revealed the statistical difference is caused by 5 patients in the delayed group with flight after diving as their probable cause for DCS compared to 0 in the early group.

### DCS treatment

Most divers in the delayed group were treated with US Navy Table 6 and 2 ATA compared to US Navy Table 6 and CX 30 in the early group (p<0.0001) (Table 2). There were no differences between the groups with respect to need for adjunctive sessions and to the number of adjunctive sessions (Table 2). It should be noted that none of the patients received any treatment, including normobaric oxygen, prior to their admission for hyperbaric treatment.

### Clinical Outcome

**Fig 2:**
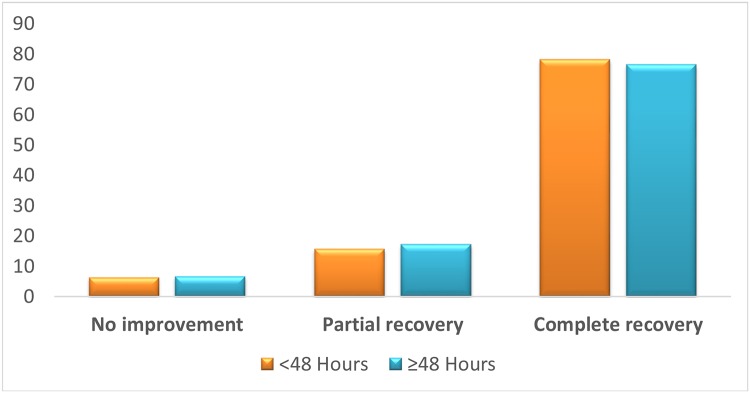
Clinical outcome of the early and delayed groups. * Clinical outcome was not significantly different between the delayed and early groups (*χ* ^2^ = 0.093, p = 0.955). Clinical outcome was divided to an ordinal variable of 3 values as shown. Graph values shown in %.

In the delayed recompression group, complete recovery was achieved in 76% of the divers, partial recovery in 17.1% and no improvement in 6.6%. It was similar to the clinical outcome of the early group where 78% of the divers had complete recovery, 15.6% had partial recovery and 6.2% had no improvement (*χ* ^2^ = 0.093, df = 2, p = 0.955) (Fig 2). In the delayed group, there was no relation between time to symptoms onset and the clinical outcome: complete recovery (74% vs. 77%), partial recovery (13% vs. 19%) and no recovery (13% vs. 4%) in divers with late vs. early symptoms onset (*χ* ^2^ = 2.438, df = 2, p = 0.296).

Analysis of the neurological DCS subset revealed no significant differences in any of the clinical outcomes of the delayed group compared to the early group: 78.7% vs. 72.4% of complete recovery, 17% vs. 19.5% of partial recovery and 4.3% vs. 8% of no improvement, (*χ* ^2^ = 0.919, df = 2, p = 0.631). Again, time to symptoms onset had no significant effect in the delayed group (*χ* ^2^ = 0.535, df = 2, p = 0.765).

There was no difference in clinical outcome between the early and the delayed group neither in divers with DCS type I (*χ* ^2^ = 3.1, df = 2, p = 0.211) nor in patients with DCS type II (*χ* ^2^ = 0.252, df = 2, p = 0.882).

### Predictors of Clinical Outcome

**Fig 3:**
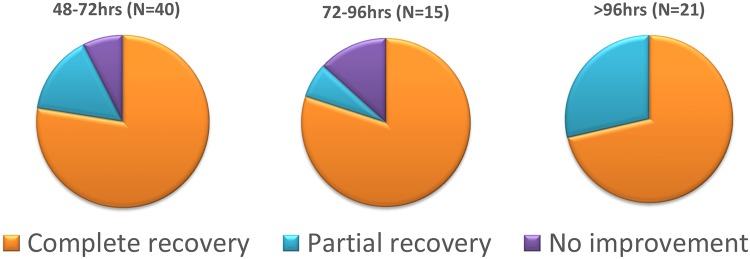
Clinical outcome and time to recompression in the delayed treatment group. * Treatment after 48, 72 and 96 hours from surfacing showed no significant differences in clinical outcome. Time to recompression was not associated with clinical outcome. Graph values shown in %.

**Fig 4:**
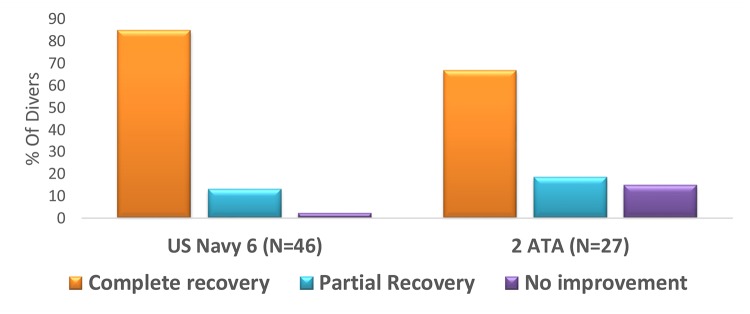
Clinical outcome and the treatment tables. * 84% and 13% of divers treated with US Navy Table 6 had complete and partial recovery compared to 66.7% and 18.5% in divers treated with 2 ATA table for 90 minutes. US Navy Table 6 had better clinical outcome than Table 2 ATA table, yet not statistically significant (*χ* ^2^ = 3.26 df = = 1 p = 0.07). Graph values shown in %.

**Fig 5:**
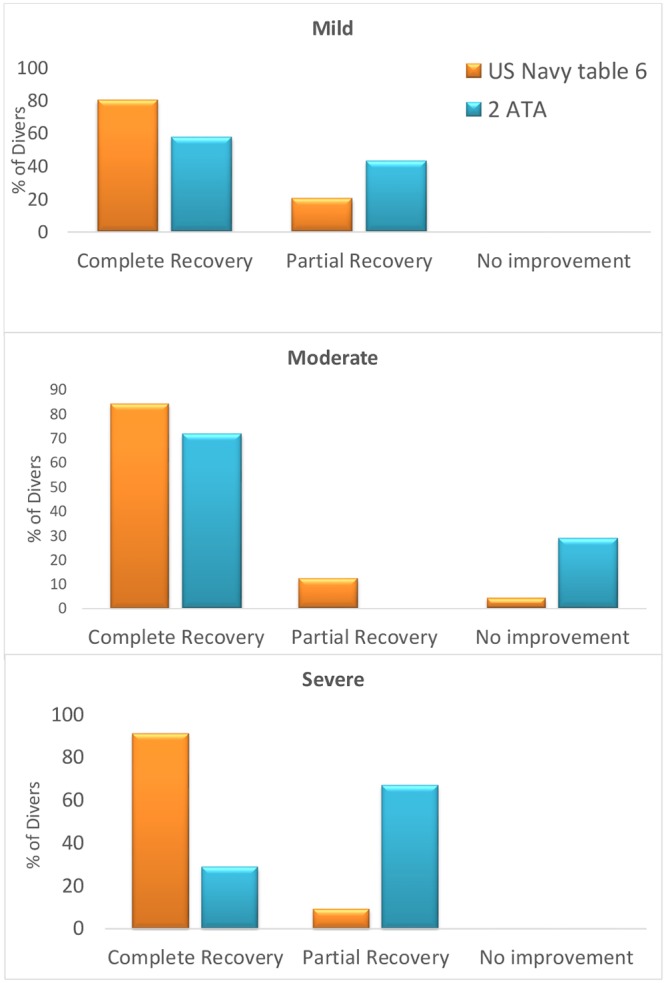
Clinical outcome by treatment table divided to severity subgroups. * Divers with moderate symptoms treated with US Navy Table 6 had 84.6% complete recovery compared to 71.4% in divers treated with 2 ATA table for 90 minutes (*χ* ^2^ = 6.26, df = 2, p = 0.04). Note US Navy Table 6 results in a trend to better outcome irrespective of severity of symptoms, yet in mild and moderate symptoms, it did not reach statistical significance.

Multivariate analysis in the delayed group revealed no significant predictor as for the clinical outcome including the following parameters: age, sex, diving experience, type of gas used during diving, probable cause of DCS, DCS type, time to recompression, time to symptoms onset, cause of DCS and severity of symptoms. The clinical outcome with relation to time to recompression in the delayed group is detailed in Fig 3. However, treatment with US Navy Table 6, compared to standard 90 minutes of HBOT, had a trend to higher percentages of full recovery in the delayed group (B = 1.025, df = 1, OR = 2.786, CI 95% [0.896–8.66], p = 0.07). As seen in Figs 4 and 5, divers treated with hyperbaric protocol according to US Navy Table 6 had better outcome compared to standard HBOT of 90 minutes, 100% oxygen at 2ATA irrespective of symptoms severity at presentation. The clinical outcome of the neurological subset in the delayed group was not related to any of the variables included in the analysis, including treatment protocol, severity, and TTR.

Multivariate analysis for all divers, from both groups, revealed that the treatment protocol was the only parameter that had statistical significant association with the clinical outcome, with US Navy Table 6 treatment having more favorable outcomes (B = 1.453, df = 1, p = 0.009, OR = 4.274, CI 95% [1.425–12.822]).

## Discussion

### Time to recompression

The study evaluated the therapeutic effect of delayed hyperbaric treatment started 48–720 hours after surfacing. The results indicate that even when delayed, hyperbaric treatment still has significant clinical value, not different from earlier treatment. The complete recovery rate, when the treatment was delayed for more than 48h, was 76% compared to 78% when treatment started earlier. This is similar to 80–86% complete recovery rates cited in the literature for early recompression [7].

The controversy regarding the beneficial effect of delayed hyperbaric treatment for DCS persists for many decades with different clinical practices used in different hyperbaric units. As it seems logical to assume that delayed treatment will result in worse prognosis, divers suffering from DCS are rushed to hyperbaric treatment and some hyperbaric units do not advise treatment for delayed cases. However, the data in the literature considering the effectiveness of delayed treatment are scarce and contradictory, as summarized in Table 4. In the classical work, done in 1964 by Rivera's, early recompression was associated with better clinical outcome mostly in DCS type 1 [10]. Kizer and Ball reported improved clinical outcome in early recompression only in small groups of severe DCS cases (26 and 24 patients respectively) [17, 18]. Van Hulst reported poor clinical outcome in a small group of 14 patients treated 24 hours or more after symptoms onset [19]. In a recent study, including 5,278 DCS cases from a fishery area in northern China, it was reported by Xu and his colleagues that complete recovery rate was higher when treatment was initiated within the first 12 hours from symptoms onset compared to those treated 24 hours after symptoms onset (91.3% vs. 79%) [7]. However, in 6 other studies, the conclusion was that time to recompression had no significant effect on the clinical outcome when proper multivariate analysis is done [5, 8, 9, 20–22].

**Table 4:** Published studies in the last decades showing the association between clinical outcome and time to recompression.

Reviews	Total Patients	Recompression > 24 hours	Significant association	Lag description
Rivera 1964	888	9	Yes in Severe cases	TTR from symptoms
Kizer 1982	50	<20	Yes	TTR from symptoms
Van Hulst 1990	121	14	Yes	TTR from symptoms
Vann 1993	1159	unknown	No	TTR from surfacing
Ball 1993	49	20	Yes in severe cases	TTR from symptoms
Boussuges 1996	96	unknown	No	TTR from surfacing
Desola 1997	466	unknown	No	TTR from symptoms
Ross 2000	360	unknown	No	TTR from symptoms
Stipp 2007	343	0	Yes	TTR from surfacing
Blatteau 2011	49	0	No	TTR from symptoms
Gempp 2010	63	0	No	TTR from symptoms
Xu 2012	5278	353	Yes	TTR from symptoms

Notice time to recompression was calculated from symptoms onset in most cases.

Most studies reports include very small cohorts, only few divers treated later than 24 hours.

What's unique to our study is the relatively large cohort of divers in whom there was significant delay in treatment greater than 48 hours. Intriguingly, there was no association between the time to recompression and the short term clinical outcome. Moreover, the delayed group didn't need excessive adjunctive hyperbaric sessions in order to achieve the same clinical efficacy as in the early group. Also, the delayed group was divided by symptoms onset assuming different pathologies, however the time lag to symptoms onset did not affect the clinical outcome as well. These findings raise an interesting question regarding the therapeutic mechanism of the hyperbaric intervention. The beneficial therapeutic effect can be either due to shrinkage of inert gas bubbles, still present in tissues even after prolonged durations of time, or it can be related to improved oxygenation of ischemic tissues.

Several reasons may account for prolonged presence of bubbles within tissues and vessels. First, in certain tissues bubbles may persist as long as the rate of nitrogen washout is disturbed. Such clearance disturbance can be mediated by blockage of the microcirculation (venous and lymphatic) by microbubbles [23]. Second, certain hematologic and immunologic active substances may generate a “shell” that surrounds the bubble. This semi-rigid "shell" may serve as a gas diffusion barrier responsible for the delay in bubble fading [24]. A third possible cause for delayed bubbles presentation can be related to the lymphatic system. The flow in the lymphatic system is relatively slow, so a significant amount of time can elapse before the lymphatic bubbles will reach the systemic circulation [25]. Fourth, micro-bubbles that form in the arterial side, have a small oxygen window (pO2 in artery—pO2 in the lungs) and therefore the driving force for inert gas elimination is limited [26].

While the recompression is delayed the occluding bubble initiates the ischemic and the ischemic/reperfusion related inflammatory cascade. The inflammatory cascade involves neutrophils migration to ischemic tissues, releasing vasoactive components, proteases and free radicals which further increase the tissue damage [14]. HBOT ameliorates the ischemic and inflammation related injuries in several ways. First, HBOT has been shown to inhibit leukocytes adhesion and activation by the damaged endothelium [11–13]. Second, HBOT may reduce the pro-inflammatory cytokine production by monocytes and macrophages[14]. Third, HBOT may improve oxygen supply to the ischemic tissue, limit the expected post-ischemic ATP depletion and decrease the expected lactate accumulation [15, 16]. In addition, as in different types of acute ischemic injuries, while necrotic tissues are not expected to recover, “penumbra” or “stunned” metabolic dysfunction areas recovery can be facilitated by HBOT [27–31].

### Optimal treatment protocol

A trend towards favorable clinical outcomes, yet not statistically significant, was noticed with US Navy Table 6, compared to hyperbaric treatment based on 90 min, 100% oxygen at 2 ATA (OR = 2.786, CI 95% [0.896, 8.66]). There might be several reasons for this observation: First, tissue oxygenation is superior while using US Navy Table 6 (240 minutes of 100% oxygen compared to 90 minutes at 2 ATA); and second, US Navy Table 6 applies 2.8 ATA compared to 2 ATA, which favors better recompression of long-standing bubbles. Since the safety profile of both tables in DCS treatment is very high, it seems reasonable to conclude that in delayed DCS, US Navy Table 6 is the preferable choice.

### Reasons for delayed treatment

All patients participating in the current study were aware of the need for early recompression once DCS is suspected. Unfortunately, a significant number of divers did not apply for professional medical care once symptoms occurred. The delay was due to personal reasons of divers hoping that the symptoms will vanish spontaneously, unclear presentation of DCS symptoms, inexperienced medical staff in primary care hospitals or difficulties of transportation to a hyperbaric institute. In our cohort, we could not find unique phenotype that characterized the delayed treatment group.

### Study limitation

The current study has several strengths and limitations. Most of the limitations are related to the fact that data was collected retrospectively and patients were not randomized to either early or delayed treatment. Obviously, from the ethical perspective, such randomized control trials cannot be applied to divers suffering from DCS since treatment should be initiated as soon possible. Retrospective cohort study may limit the effective use of Odds ratio analysis and may increase the risk for selection bias. In order to eliminate this risk, all patients treated for DCS in our institute since its establishment were included without any selection. The fact that clinical outcome was evaluated 10–14 days after treatment is another limitation since the proportion of divers with long term sequel may change in a longer follow-up.

With regards to strengths, a series of 76 patients treated 48 hours after surfacing is one of the largest cohorts of delayed treatment reported. However, further studies are needed by other hyperbaric centers for optimization of the treatment protocol.

## Conclusions

Late hyperbaric treatment for DCS, 48 hours or more after surfacing, has a significant clinical value and can achieve complete recovery in 76% of the divers. It seems that the preferred protocol should be based on US Navy Table 6. Further studies focusing on this cohort of divers, referring late for medical assistance, are needed in order to optimize their management.
